# Associations of health, physical activity and weight status with motorised travel and transport carbon dioxide emissions: a cross-sectional, observational study

**DOI:** 10.1186/1476-069X-11-52

**Published:** 2012-08-03

**Authors:** Anna Goodman, Christian Brand, David Ogilvie

**Affiliations:** 1 Faculty of Epidemiology and Population Health, London School of Hygiene and Tropical Medicine, Keppel Street, London WC1E 7HT, UK; 2 Environmental Change Institute and Transport Studies Unit, Oxford University Centre for the Environment, Oxford University, South Parks Road, Oxford OX1 3QY, UK; 3 Medical Research Council Epidemiology Unit & UKCRC Centre for Diet and Activity Research (CEDAR), Institute of Public Health, Box 296, Forvie Site, Robinson Way, Cambridge CB2 0SR, UK

**Keywords:** Carbon emissions, Active travel, Health, Motor vehicles, Physical activity, Weight

## Abstract

**Background:**

Motorised travel and associated carbon dioxide (CO_2_) emissions generate substantial health costs; in the case of motorised travel, this may include contributing to rising obesity levels. Obesity has in turn been hypothesised to increase motorised travel and/or CO_2_ emissions, both because heavier people may use motorised travel more and because heavier people may choose larger and less fuel-efficient cars. These hypothesised associations have not been examined empirically, however, nor has previous research examined associations with other health characteristics. Our aim was therefore to examine how and why weight status, health, and physical activity are associated with transport CO_2_ emissions.

**Methods:**

3463 adults completed questionnaires in the baseline iConnect survey at three study sites in the UK, reporting their health, weight, height and past-week physical activity. Seven-day recall instruments were used to assess travel behaviour and, together with data on car characteristics, were used to estimate CO_2_ emissions. We used path analysis to examine the extent to which active travel, motorised travel and car engine size explained associations between health characteristics and CO_2_ emissions.

**Results:**

CO_2_ emissions were higher in overweight or obese participants (multivariable standardized probit coefficients 0.16, 95% CI 0.08 to 0.25 for overweight vs. normal weight; 0.16, 95% CI 0.04 to 0.28 for obese vs. normal weight). Lower active travel and, particularly for obesity, larger car engine size explained 19-31% of this effect, but most of the effect was directly explained by greater distance travelled by motor vehicles. Walking for recreation and leisure-time physical activity were associated with higher motorised travel distance and therefore higher CO_2_ emissions, while active travel was associated with lower CO_2_ emissions. Poor health and illness were not independently associated with CO_2_ emissions.

**Conclusions:**

Establishing the direction of causality between weight status and travel behaviour requires longitudinal data, but the association with engine size suggests that there may be at least some causal effect of obesity on CO_2_ emissions. More generally, transport CO_2_ emissions are associated in different ways with different health-related characteristics. These include associations between health goods and environmental harms (recreational physical activity and high emissions), indicating that environment-health ‘co-benefits’ cannot be assumed. Instead, attention should also be paid to identifying and mitigating potential areas of tension, for example by promoting low-carbon recreational physical activity.

## Background

Climate change poses profound threats to human health, including via extreme weather events, infectious diseases, food insecurity and broader social disruptions [1]. A major driver of climate change is global energy use and the associated production of greenhouse gases, particularly carbon dioxide (CO_2_) [2]. The transport sector generates around a quarter of these greenhouse gas emissions (23% globally [3], 27% in the United Kingdom (UK) [2]), and this contribution is rising in both absolute and relative terms [3].

Motorised travel also adversely affects current health by increasing urban air pollution, noise pollution and road traffic crashes [4]. Motorised travel — particularly car travel — may also contribute to rising obesity levels, both because it is a sedentary behaviour itself and because it may displace physically active modes such as walking or cycling. In recent years, studies from around the world have documented a positive cross-sectional association between car use and overweight or obesity [5-8] and one Chinese study has also demonstrated a prospective association [9].

Yet while a causal effect linking car use to obesity is highly plausible, a recent paper hypothesised that obesity may also increase motorised travel and/or CO_2_ emissions [10]. First, heavier people may *use motorised travel more* because, particularly at higher weights, walking requires greater physical effort. Secondly, heavier people may *choose larger cars* and so increase their fuel consumption. In mathematical models, these pathways both contributed approximately a 12% increase in transport CO_2_ emissions from a population with an ‘overweight’ versus a ‘normal’ weight distribution. This led the authors to conclude that the obesity epidemic is an important environmental problem as well as a public health priority [10]. To our knowledge, however, the modelling assumption that heavier individuals choose larger cars has never been tested empirically, nor has any study examined whether weight status is in fact associated with transport CO_2_ emissions. Empirical examination is particularly warranted because overweight and obesity are also major contributors to ill health [11]. This poorer health status could plausibly have effects in either direction, potentially strengthening the association by further diminishing the ability to walk or cycle, or alternatively decreasing the association by diminishing the ability to travel at all.

As for associations with physical activity, it is widely assumed that walking or cycling for transport (‘active travel’) substitutes for at least some motorised travel and thereby reduces CO_2_ emissions [e.g. [12]. This assumption is supported by the finding that energy expenditure from walking is negatively correlated with fossil fuel use from car driving [13] and that individuals in ‘walkable’ neighbourhoods make more walking trips and travel fewer vehicle miles [14]. One study has also reported that lower car use is associated with increased likelihood of meeting recommendations for total physical activity [5]. For these reasons, promoting active travel has been discussed as one area in which measures undertaken to reduce greenhouse gas emissions may also produce health ‘co-benefits’ [15,16]. However, we know of no previous study which has disaggregated total physical activity and examined the carbon implications of engaging in recreational walking, cycling or other physical activity.

The lack of empirical evidence on these issues partly reflects the fact that studies estimating CO_2_ emissions do not usually collect ‘health’ data, and vice versa. This paper therefore capitalises upon a unique interdisciplinary study to examine how and why health, physical activity and weight status are associated with transport CO_2_ emissions. In doing so, we test the general hypothesis that any associations may in part be explained by differences in levels of active and/or motorised travel. We also test the more specific hypothesis that obese individuals generate more CO_2_ emissions because they use larger cars.

## Methods

### Study population

Our analyses use baseline cross-sectional data from the iConnect study, an observational ‘natural experimental’ study seeking to examine the effects of new transport infrastructure on travel, physical activity and CO_2_ emissions [17,18]. Briefly, 22,500 adults in three areas of the UK (Cardiff, Kenilworth and Southampton) were randomly selected from the edited electoral register in April 2010 and sent questionnaires and consent forms by post. A blank copy of the questionnaire is included in Additional file 1; the raw data cannot be made freely available because they contain confidential, potentially-identifiable information. The University of Southampton Research Ethics Committee granted ethical approval (CEE200809-15).

In total, 3516 individuals returned questionnaires (a 16% response rate). In our analyses we excluded participants who did not report any travel in the past week (n = 42), who provided invalid physical activity data (n = 4), or who were missing data on more than half of our covariates (n = 7). The resulting study population comprised 3463 individuals (age range 18–96, 45% male). Comparisons with local and national data suggested that participants in our study population were somewhat healthier than the general population and were also less likely to live in a household with children and more likely to have a degree. Otherwise, however, our study population appeared to be broadly similar in terms of its demographic, socio-economic and travel-related characteristics (see Additional file 2 for full details).

### CO_2_ emissions, travel distance and car size

A detailed seven-day recall instrument assessed travel for five journey purposes: commuting for work, commuting for education, travel in the course of business, shopping or personal business, and social visits or leisure activities. For each journey purpose, participants reported the total number of journeys made, plus the total time spent and distance travelled by seven modes: walking, cycling, bus, train, car (as a driver), car (as a passenger) and ‘other’. If only distance or time was reported then the counterpart was imputed using the mean observed speed for each combination of mode and journey purpose (see Additional file 3).

We used these travel diary data to derive the total distance travelled in the past week by active travel (walking and cycling) and by motorised modes (bus, train, car, and other motorised modes). As described fully in Additional file 3, we also derived transport CO_2_ emissions by multiplying the distance travelled by each motorised mode by that mode’s average emissions factors [19]. For travel by cars we additionally calibrated these emissions factors by (1) using the number of trips to take into account excess emissions following ‘cold’ engine starts and (2) applying speed-emissions factors [20] based on average speed plus the self-reported fuel type, engine size and age of the car the participant used most. As we lacked detailed data on car-sharing we modelled CO_2_ in two ways, one assigning all emissions to the driver and one dividing emissions between passengers and drivers. Our substantive findings were generally identical and we therefore report results using the second approach (see Additional file 3 for further discussion).

Other outcomes of interest were the engine size of the participant’s main car (response options: <1.4 litres, 1.4-2 litres, >2 litres) and the car size. We assigned car size by using make and model to classify cars according to the UK’s Motor Vehicle Registration Information System ( http://www.smmt.co.uk). We then created four categories based upon typical interior volume: small (minis, superminis and sport coupés), medium (medium cars), large (large cars, executive cars, luxury cars) and very large (sports utility vehicles, multi-purpose vehicles, vans and pick-up trucks).

### Health, body mass index and physical activity

Participants reported their general health (response options: excellent, good, fair, poor) and whether they had a long-term illness, health problem or disability which limited daily activities (response options: yes, no). They also provided self-reported height and weight, from which we calculated body mass index (kg/m^2^). Applying standard cut-offs, we classified participants as being of normal weight (body mass index (BMI) < 25), overweight (25 ≤ BMI < 30) or obese (30 ≤ BMI). We measured recreational physical activity using four items adapted from the short form of the International Physical Activity Questionnaire [21]. Participants reported the number of sessions and total time spent in the past week in walking for recreation, cycling for recreation, moderate physical activity and vigorous physical activity. We combined these last two variables into a single measure of other leisure-time moderate-to-vigorous physical activity (MVPA), and excluded participants reporting more than 16 hours per day in any of our four activity measures. We used the seven-day travel instrument described above to measure past-week time spent walking and cycling for transport and distance covered walking and cycling for transport.

### Socio-demographic and environmental covariates

Table 1 presents the socio-demographic and environmental variables which we included as covariates in multivariable analyses; all were self reported except for study site and urban/rural status which were assigned from home postcode. All groupings were determined *a priori*, reflecting a desire to achieve groups of equal width (e.g. for age) and/or with a minimum sample size of at least 100 (e.g. ethnicity). We divided covariates into ‘potential confounders’ and ‘potential confounders or mediators’, the latter being variables which we hypothesised might sometimes mediate an association between health characteristics and CO_2_ emissions (e.g. economic inactivity and income loss because of poor health status, or buying an extra car because obesity makes walking difficult).

**Table 1:** Socio-demographic and environmental characteristics of participants

**Conceptual domain**	**Variable**	**Level**	**n (%)**
Potential confounders	Sex	Female	1900 (55%)
		Male	1556 (45%)
	Age (years)	18-34	791 (23%)
		35-49	801 (23%)
		50-64	991 (29%)
		>65	836 (24%)
	Ethnicity	White	3239 (95%)
		Non-White	178 (5%)
	Any child under 16	No	2715 (79%)
		Yes	701 (21%)
	Highest educational qualification	Degree	1372 (41%)
		A-level or equivalent	599 (18%)
		GCSE or equivalent	629 (19%)
		None or other	756 (23%)
	Site	Southampton	1108 (32%)
		Cardiff	1109 (32%)
		Kenilworth	1246 (36%)
	Urban/rural status	Urban	3305 (95%)
		Rural	158 (5%)
Potential confounders or mediators	Employment status	Full-time	1403 (41%)
		Part-time	475 (14%)
		Student	222 (7%)
		Retired	937 (28%)
		Home duties	145 (4%)
		Sick/unemployed/other	212 (6%)
	Annual household income	>£40,000	1056 (37%)
		£20,001-40,000	934 (33%)
		≤£20,000	876 (31%)
	Cars per adult in household	No cars	504 (15%)
		<1 car per adult	1282 (37%)
		≥1 cars per adult	1638 (48%)
	Any adult bike in household	No	1375 (42%)
		Yes	1883 (58%)

n < 3463 in some variables because of missing data. See Table 2 for health characteristics.

### Statistical analysis

The percentage of missing data for our explanatory variables and covariates ranged from 0 to 17%. We used multiple imputation by chained equations (five imputations) to impute missing values under an assumption of missing at random, including in the imputation model all covariates and outcomes ever entered into the regression models. Because response rates differed somewhat by age and sex (see Additional file 2), we weighted participants by the 2001 age and sex profile of their Lower Super Output Area (administrative areas with a population of around 1500) [22]. All analyses used Stata11 except for the path analysis which used MPlus5.

We used linear regression to examine the predictors of total transport CO_2_ emissions and CO_2_ emissions by journey type. We entered variables categorically as all showed evidence of non-linear associations with CO_2_ (all p < 0.05 and most p < 0.001 for non-linearity in univariable analyses, as judged by including a quadratic term). We initially used a hierarchical approach to build multivariable models, first adjusting for our ‘potential confounder’ covariates (entered categorically, as shown in Table 1), then adjusting for our ‘potential confounder or mediator’ covariates, and finally adjusting for time in active travel. Because CO_2_ emissions were positively skewed, we applied the transformation ‘log([x/mean(x)] + 0.01)’ (adding 0.01 to avoid turning zeros into missing values) and then standardised these log-transformed outcomes. We fitted single-level regression models because fitting multi-level models indicated that spatial clustering was low (2.9% variation in log-transformed CO_2_ at site level) and did not affect our substantive findings.

**Figure 1:**
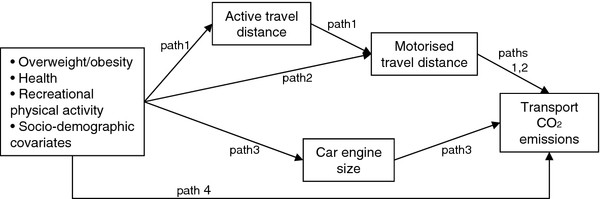
**Path analysis examining the contribution of active travel, motorised travel and engine size.** Path 1 = indirect (mediated) path via active + motorised travel; path 2 = indirect path via motorised travel only; path 3 = indirect path via car engine size; path4 = direct path (‘unexplained’).

We next examined the predictors of car size and car engine size using ordered logistic regression. Finally we examined the extent to which the observed associations with total CO_2_ emissions could be explained by motorised travel, active travel and/or engine size. To do this we fitted the model shown in Figure 1 using path analysis, that is by fitting a structural equation model containing no latent variables [23]. Because one of our mediators (engine size) was ordinal, we fitted this model using probit regression with the weighted least squares estimator. Note that although Figure 1 assumes that motorised travel, active travel and car engine size are consequences of our health and activity variables (i.e. mediators of the association between health and CO_2_ emissions), the cross-sectional nature of our data means we cannot examine statistically the extent to which they may instead be causes (i.e. confounders of the association between health and CO_2_ emissions). Our path analysis did, however, allow us to establish which of these three potential mediator/confounder variables were most important in explaining the overall association between our health/activity variables and CO_2_ emissions.

## Results

### Predictors of transport CO_2_ emissions

Our participants generated an estimated median of 18.8 kilograms of transport CO_2_ in the past week (mean 35.1, interquartile range 6.2 to 42.0). These emissions were distributed highly unequally: the top fifth of participants generated 63% of the total while the bottom fifth generated just 0.8%. Car driving was by far the single largest source of emissions (89.8%), followed by train travel (4.4%), bus travel (3.8%), and other private or public transport (1.9%).

As shown in Table 2, there was strong evidence that overweight status was associated with higher total CO_2_ emissions, with log-transformed emissions among overweight individuals being 0.25 standard deviations higher than among normal weight individuals after adjusting for potential confounders (95% CI 0.16, 0.34: multivariable model 1). There was a very similar effect size for obesity after adjusting for the fact that obese people reported worse general health (41% reported fair or poor health vs. 16% of participants of normal weight). These effects were somewhat attenuated after adjusting for car access (model 2) and time spent in active travel (model 3), but there remained strong evidence of an independent effect (p < 0.001 for heterogeneity).

**Table 2:** **Health and physical activity predictors of transport CO**_**2**_**emissions (n = 3463)**

**Variable**	**Level**	**n†(%)**	**Median kgCO**_**2**_**/**	**Linear regression coefficients and 95%CI for total standardised log-transformed CO**_**2**_**emissions**			
			**week**	**Minimally-adjusted**	**Multivariable model 1**	**Multivariable model 2**	**Multivariable model 3**
Weight status	Normal	1679 (51%)	16.6	0***	0***	0***	0**
	Overweight	1140 (35%)	23.4	.22 (.12, .32)	.25 (.16, .34)	.16 (.08, .24)	.14 (.06, .22)
	Obese	472 (14%)	20.3	.09 (−.05, .23)	.23 (.10, .37)	.16 (.04, .28)	.14 (.02, .25)
General health	Excellent/good	2664 (78%)	20.7	0***	0***	0	0
	Fair	639 (19%)	13.9	-.24 (-.35, -.12)	-.15 (-.26, -.04)	-.05 (-.15, .04)	-.06 (-.15, .04)
	Poor	126 (4%)	6.1	-.69 (-.87, -.50)	-.47 (-.67, -.27)	-.16 (-.35, .04)	-.17 (-.36, .01)
Long-term illness	No	2553 (78%)	21.0	0***	0*	0	0
	Yes	727 (22%)	12.9	-.34 (-.46, -.23)	-.15 (-.26, -.03)	.03 (-.07, .14)	.02 (-.08, .12)
Walking for recreation in past week	None	1394 (40%)	15.3	0***	0**	0***	0***
	1-149 min	1119 (32%)	21.1	.28 (.18, .38)	.18 (.08, .27)	.16 (.07, .24)	.16 (.08, .24)
	150-419 min	663 (19%)	20.5	.28 (.17, .40)	.17 (.06, .28)	.19 (.10, .28)	.22 (.12, .31)
	≥420 min	287 (8%)	21.2	.13 (-.05, .32)	.10 (-.07, .27)	.14 (-.02, .30)	.15 (-.01, .31)
Cycling for recreation in past week	None	3018 (87%)	18.3	0	0**	0*	0
	1-149 min	287 (8%)	20.1	-.09 (-.27, .09)	-.23 (-.40, -.06)	-.14 (-.30, .01)	.02 (-.14, .18)
	≥150 min	158 (5%)	21.6	-.24 (-.51, .03)	-.35 (-.61, -.09)	-.22 (-.44, .00)	.06 (-.17, .28)
Other leisure-time MVPA in past week	None	1521 (44%)	13.6	0***	0***	0***	0***
	1-149 min	781 (23%)	21.6	.35 (.24, .45)	.22 (.12, .32)	.15 (.06, .24)	.16 (.07, .25)
	150-419 min	806 (23%)	22.4	.46 (.36, .56)	.33 (.23, .43)	.23 (.14, .32)	.23 (.15, .32)
	≥420 min	355 (10%)	26.7	.34 (.16, .53)	.22 (.05, .39)	.18 (.03, .33)	.17 (.02, .31)
Walking for transport in past week	None	1231 (36%)	24.7	0***			0***
	1-149 min	1245 (36%)	19.7	-.22 (-.32, -.12)			-.10 (-.18, -.02)
	150-419 min	723 (21%)	14.1	-.49 (-.60, -.38)			-.22 (-.32, -.12)
	≥420 min	264 (8%)	10.0	-.70 (-.90, -.50)			-.22 (-.40, -.04)
Cycling for transport in past week	None	3042 (88%)	19.9	0***			0***
	1-149 min	253 (7%)	12.7	-.51 (-.69, -.33)			-.40 (-.57, -.23)
	≥150 min	168 (5%)	9.0	-.73 (-.96, -.49)			-.59 (-.82, -.36)

*p < 0.05,**p < 0.01,***p < 0.001 for heterogeneity. *CI* = confidence interval, *kgCO*_*2*_ = kilograms carbon dioxide, *MVPA* = moderate-to-vigorous physical activity. †n < 3463 in some variables because of missing data; multiple imputation used to include all participants in regression models. Minimally-adjusted analyses adjust for age and sex, model 1 adjusts for variables in column plus ‘potential confounders’ in Table 1; models 2 and 3 adjust for variables in column plus all variables (‘confounders’ and ‘mediators’) in Table 1.

By contrast, despite the association between obesity and poorer general health, CO_2_ emissions were lower among participants reporting poorer general health or a long-term illness. This effect was attenuated to the null in model 2, however, after adjusting for the fact that poor health was associated with economic inactivity and lower car access (e.g. 45% of those with fair or poor general health were sick or retired and 27% had no car in their household, vs. 26% and 11% respectively for those with excellent or good health).

As for physical activity, there was strong evidence that time spent in recreational walking or other leisure-time MVPA was positively associated with CO_2_ emissions. This was observed across all multivariable analyses, and subdividing emissions by journey purpose indicated that the effect sizes were largest for social or leisure travel (model 3 regression coefficients 0.12 to 0.31 for social or leisure travel vs. 0.01 to 0.18 for other purposes; see Additional file 4). Recreational cycling showed a different pattern, with an initial negative association that was attenuated to the null after adjusting for past-week cycling for transport. Finally, there was strong evidence of an approximately dose–response relationship between increasing time spent in walking or cycling for transport and decreasing transport CO_2_ emissions.

### Predictors of car size and car engine size

Among the 2792 participants who reported ever travelling by car in the last week, obesity was associated with larger car size (proportional odds ratio (OR) 1.48, 95% CI 1.13, 1.96 for obese vs. normal weight in minimally-adjusted analyses; OR 1.50, 95% CI 1.13, 21.98 after adjusting for the covariates in Table 1 plus the total distance travelled by car). This reflected the fact that obese individuals were less likely to have a small car (33% vs. 36% of overweight and 44% of normal-weight individuals) and more likely to have a very large car (16% vs. 12% of overweight and 10% of normal-weight individuals). There was likewise evidence of an association between obesity and car engine size (minimally-adjusted OR 1.37, 95% CI 1.08, 1.75; multivariable OR 1.36, 95% CI 1.06, 1.74), an association which was attenuated to the null after additionally adjusting for car size (OR 1.13, 95% CI 0.85, 1.50). This therefore supported our hypothesis that obesity would be associated with larger car size and, for this reason, with larger car engine size.

As for overweight, this showed a trend in the same direction but the effect sizes were smaller and non-significant (multivariable OR 1.18, 95% CI 0.98, 1.42 for overweight vs. normal weight with respect to car size; OR 1.08, 95% CI 0.88, 1.33 for engine size). No other health or physical activity variable was associated with either car size or engine size in minimally-adjusted or multivariable analyses (all p ≥ 0.1).

### Relative contributions of active travel, motorised travel and engine size

Table 3 presents the associations between our health and physical activity variables and the distances travelled by active travel, by motorised travel and overall. Table 4 presents the results of fitting the path analysis shown in Figure 1. As shown in Table 4, there was strong evidence that the excess CO_2_ emissions associated with overweight or obesity were partly explained by reduced active travel distance (path 1; see also Table 3) and, in the case of obesity, by larger engine size (path 3). Nevertheless the magnitudes of these ‘active travel’ and ‘engine size’ paths were relatively small compared to the total effects, together explaining only 19% of the effect for overweight and 31% of the effect for obesity. Instead the majority of the effect resulted from the fact that, even adjusting for differences in active travel distance, overweight and obese individuals travelled a greater distance by motorised modes (path 2). This reflected the fact that the increase in motorised travel distance was larger than the decrease in active travel distance, resulting in a greater total travel distance among overweight or obese individuals (see Table 3). As was the case for all the health and activity variables, there was no evidence of any residual direct association between overweight or obesity and CO_2_ emissions (Table 4, path 4), indicating that the specified indirect pathways explained the observed total associations.

**Table 3:** Health and physical activity predictors of past-week distance travelled by active modes, motorised modes and overall (n = 3463)

		**Active travel distance**		**Motorised travel distance**		**Total travel distance**	
		**Median km/week**	**Multivariable regression coefficient (95%CI)**	**Median km/week**	**Multivariable regression coefficient (95%CI)**	**Median km/week**	**Multivariable regression coefficient (95%CI)**
Total sample		4.8		128.1		132.9	
Weight status	Normal	6.4	0***	117.5	0***	131.9	0**
	Overweight	3.2	-.17 (-.25, -.08)	148.8	.15 (.07, .24)	160.9	.12 (.04, .19)
	Obese	1.7	-.16 (-.27, -.06)	133.6	.16 (.04, .27)	149.6	.12 (.01, .23)
General health	Excellent/good	4.8	0*	141.0	0	154.5	0*
	Fair	3.2	-.10 (-.20, -.01)	95.9	-.06 (-.16, .04)	106.2	-.09 (-.19, .01)
	Poor	0.8	-.18 (-.36, .00)	42.9	-.15 (-.35, .05)	54.6	-.28 (-.49, -.06)
Long-term illness	No	5.8	0*	143.2	0	155.0	0
	Yes	1.6	-.13 (-.24, -.02)	9.1	.03 (-.08, .13)	96.5	-.02 (-.13, .09)
Walking for recreation in past week	None	3.0	0***	103.3	0***	115.4	0***
	1-149 min	6.4	.17 (.08, .25)	141.6	.17 (.08, .25)	153.9	.17 (.09, .25)
	150-419 min	6.4	.25 (.15, .35)	144.7	.19 (.10, .29)	157.7	.24 (.16, .33)
	≥420 min	7.8	.32 (.18, .46)	15.8	.17 (.00, .33)	173.0	.31 (.19, .43)
Cycling for recreation in past week	None	3.7	0***	123.1	0*	133.9	0
	1-149 min	14.5	.29 (.15, .42)	157.7	-.14 (-.30, .02)	175.4	-.05 (-.19, .09)
	≥150 min	24.9	.56 (.38, .75)	148.8	-.21 (-.44, .02)	210.1	.09 (-.08, .25)
Other leisure-time MVPA in past week	None	3.2	0	94.9	0***	103.7	0***
	1-149 min	5.5	.09 (.00, .18)	149.6	.16 (.06, .25)	161.7	.20 (.11, .29)
	150-419 min	6.4	.09 (-.01, .18)	148.0	.23 (.14, .32)	164.1	.22 (.14, .31)
	≥420 min	5.6	.01 (-.13, .14)	177.0	.18 (.03, .33)	191.5	.23 (.11, .35)

*p < 0.05,**p < 0.01,***p < 0.001 for heterogeneity. *CI* = confidence interval, *MVPA* = moderate-to-vigorous physical activity. Analyses conducted using linear regression, adjusting for all variables in columns and in Table 1 (equivalent to multivariable model 2 in Table 2). Outcomes for regression analyses are standardised log-transformed distances.

**Table 4:** **Examining the contributions of motorised travel distance, active travel distance and engine size (n = 3463), fitting model in Figure**1

		**Multivariable probit regression coefficients and 95%CI for effects on total standardised log-transformed transport CO**_**2**_**emissions**				
		**Indirect via active + motorised travel (path 1)**	**Indirect via motorised travel only (path 2)**	**Indirect via engine size (path 3)**	**Direct (‘unexplained’) (path 4)**	**Total (sum of paths 1–4)**
Weight status	Normal	0	0	0	0	0
	Overweight	.022 (.010,.034)	.124 (.042,.205)	.008 (-.007,.024)	.007 (-.017,.031)	.161 (.077,.245)
	Obese	.022 (.007,.036)	.127 (.011,.243)	.028 (.004,.051)	-.016 (-.049,.017)	.161 (.043,.279)
General health	Excellent/good	0	0	0	0	0
	Fair	.014 (.000,.028)	-.070 (-.160,.020)	-.004 (-.025,.016)	.008 (-.021,.036)	-.052 (-.145,.041)
	Poor	.024 (-.006,.053)	-.171 (-.362,.020)	-.018 (-.062,.027)	.011 (-.044,.067)	-.153 (-.341,.035)
Long-term illness	No	0	0	0	0	0
	Yes	.017 (.003,.031)	.007 (-.094,.109)	-.004 (-.026,.018)	.014 (-.017,.044)	.034 (-.069,.137)
Walking for recreation in past week	None	0	0	0	0	0
	1-149 min	-.022 (-.036,-.008)	.182 (.101,.263)	-.007 (-.024,.011)	.002 (-.024,.027)	.155 (.071,.240)
	150-419 min	-.033 (-.048,-.017)	.217 (.123,.312)	.019 (−.003,.040)	-.016 (-.046,.014)	.188 (.090,.286)
	≥420 min	-.042 (-.062,-.022)	.198 (.072,.324)	.016 (-.011,.044)	-.033 (-.069,.003)	.139 (.008,.270)
Cycling for recreation in past week	None	0	0	0	0	0
	1-149 min	-.037 (-.058,-.016)	-.094 (-.215,.026)	.001 (-.024,.026)	-.014 (-.048,.019)	-.145 (-.271,-.018)
	≥150 min	-.074 (-.103,-.045)	-.125 (-.282,.031)	-.010 (-.047,.027)	-.014 (-.052,.025)	-.223 (-.383,-.062)
Other leisure-time MVPA in past week	None	0	0	0	0	0
	1-149 min	-.012 (−.024,.000)	.161 (.075,.247)	-.001 (-.018,.017)	.004 (-.022,.029)	.152 (.061,.243)
	150-419 min	-.011 (-.023,.001)	.231 (.135,.327)	-.001 (-.020,.019)	.007 (-.022,.036)	.227 (.128,.325)
	≥420 min	-.001 (-.017,.015)	.168 (.039,.297)	.020 (-.008,.048)	-.007 (-.044,.030)	.181 (.047,.315)

*CI* = confidence interval, *MVPA* = moderate-to-vigorous physical activity. Analyses adjust for all variables in columns and in Table 1. Endogenous distance and CO_2_ variables are standardised log-transformed. Note that the ‘total effects’ in this table are equivalent to the results shown in multivariable model 2 of Table 2, except that these are probit rather than linear regression coefficients.

A different pattern was seen for recreational walking and other leisure-time MVPA, which were positively associated with both active travel distance and motorised travel distance (albeit with marginal significance for leisure-time MVPA: see Table 3). The indirect ‘active travel’ path therefore contributed to a reduction in total CO_2_ emissions in both variables (path 1 regression coefficients −0.001 to −0.042 across the two variables). The magnitude of this negative effect was more than offset, however, by the much larger positive association with motorised travel (path 2 regression coefficients 0.161 to 0.231). Thus for both variables, the increased motorised travel distance (and associated CO_2_ emissions) occurred *in spite of* rather than because of changes in active travel distance. By contrast, the increased active travel associated with recreational cycling did seem to displace an equivalent amount of motorised travel distance (Table 3), leading to an overall negative association with CO_2_ emissions (Table 4, final column).

## Discussion

In this study both overweight and obesity were independently associated with higher transport CO_2_ emissions. This was partly explained by reduced active travel and larger car size, but was mostly explained by increased motorised travel distance (even after adjusting for reduced active travel). Walking for recreation and other leisure-time physical activity were likewise both independently associated with greater motorised travel and higher CO_2_ emissions, thereby contrasting with walking or cycling for transport which were both associated with lower CO_2_ emissions.

### Strengths and limitations

In interpreting these findings it is important to bear in mind this study’s limitations. Our 16% response rate means that our sample cannot be assumed to be representative and our results cannot be assumed to be generalisable. A second key limitation is the cross-sectional nature of our data, meaning that the direction of causality (if any) behind many of the observed associations is unclear. Third, although the interdisciplinarity of the iConnect study is what made this paper possible, it also meant that we measured travel, physical activity and health using relatively brief instruments. In combination with the self-reported nature of our height, weight and physical activity data, this is likely to have introduced some measurement error and may therefore have attenuated our estimates of the effect sizes. Finally, despite our adjustment for a range of demographic, socio-economic and environmental characteristics, there remains (as in all observational studies) the possibility of unmeasured or residual confounding.

Yet while replication of these findings is therefore needed, our study population appeared to be broadly similar to the general population, albeit somewhat healthier and better educated. Taken in conjunction with the consistency of our findings with those of previous research examining the links between obesity and motorised travel [5-9], this provides some reason to believe that our findings do not arise simply from bias. With one exception [9], however, these previous studies were cross-sectional, underlining the need for further longitudinal studies. Previous studies have also been largely confined to the associations between obesity, active travel and motorised travel [5-9,13]. By contrast, the interdisciplinary scope of our study enabled us to examine, for the first time, the association between health and physical activity (measured on a number of dimensions) and CO_2_ emissions (including via measurement of both travel behaviour and vehicle characteristics).

### Interpretation of findings and directions for future research

Beyond simply documenting these associations between health characteristics and CO_2_ emissions, our study has also quantified the relative contribution of different hypothesised mechanisms and provided some clues as to the likely directions of causality. For example, we found that the association between overweight/obesity and CO_2_ emissions was primarily explained by greater motorised travel distance. We also report the novel finding that overweight or obese individuals travelled further *in total* (i.e. summing motorised and active modes). Although longitudinal data are required to confirm this, it seems more plausible to us that weight gain may be a result of desiring or needing to travel further (e.g. because of work or family commitments) rather than a cause of increased travel. If so, this would suggest that the association between overweight/obesity and CO_2_ emissions primarily reflects confounding by motorised travel distance rather than a forward causal effect of obesity.

Less clear is the direction of causality underlying the association of overweight and obesity with reduced active travel and with higher car ownership: causal effects in both directions seem plausible and longitudinal data are therefore required to establish their relative magnitudes. Nevertheless, at least some forward causal effect of obesity upon CO_2_ emissions is suggested by our novel empirical demonstration that obese and perhaps overweight individuals had larger cars (an association which we judge unlikely to reflect reverse causality). It is, however, worth highlighting that the contribution of this pathway was relatively small, and that the observed association between weight and car size was substantially weaker than the assumption in a previous modelling paper that all normal or overweight individuals would use a small car and all obese individuals would use a very large car [10].

Another original finding was that recreational walking and other leisure-time MVPA were strongly associated with increased motorised travel distance and CO_2_ emissions. Since both variables were also associated with increased active travel, we believe this association does not simply reflect activity substitution (e.g. driving to work because one is tired after playing tennis). Instead these recreational activities were associated with greater *total* travel distance, suggesting that they may have entailed additional (predominantly motorised) trips – for example, driving to a tennis court or to the start of a walking route. This hypothesis is supported by the fact that these recreational activities were most strongly associated with CO_2_ emissions from social or leisure journeys. Further research is warranted to test this hypothesis more thoroughly (e.g. using trip-level travel diary data) and, if substantiated, to examine ways to promote and facilitate low-carbon forms of recreational physical activity. Recreational cycling appeared one such low-carbon activity, albeit one which was not frequently practised in this population (past-week prevalence 12%). Walking and cycling for transport were strongly negatively associated with CO_2_ emissions, thereby bolstering the case for considering active travel to be a particularly environmentally-friendly means of meeting physical activity recommendations [e.g. [13].

## Conclusions

Transport CO_2_ emissions are associated in different ways with different health-related characteristics. This includes instances where health and environmental ‘goods’ are associated (active travel and low emissions); where health and environmental harms are associated (overweight or obesity and high emissions); and where a health good is associated with an environmental harm (recreational physical activity and high emissions). Although further longitudinal analysis is warranted to clarify the direction of causality underlying some of these associations, this work highlights that ‘co-benefits’ [15] cannot be assumed. Instead, attention should also be paid to identifying and mitigating potential areas of tension. Studying health and the environment simultaneously can thereby inform policies to address the twin goals of improving public health and promoting environmental sustainability.

## Abbreviations

BMI: Body mass index; CI: Confidence interval; CO2: Carbon dioxide; MVPA: Moderate-to-vigorous physical activity; OR: Odds ratio; UK: United Kingdom.

## Competing interests

The authors declare that they have no competing interests.

## Authors’ contributions

DO leads the iConnect work package that includes this survey, and DO and CB participated in the design of the survey. AG and CB defined the research questions addressed in this paper, with CB calculating carbon emissions and AG performing statistical analyses. AG drafted the manuscript, and CB and DO revised it critically for important intellectual content. All authors read and approved the final manuscript.

## Supplementary Material

Additional file 1 ‘Survey Questionnaire’: Blank copy of survey questionnaire. Click here for file

Additional file 2 ‘Representativeness of study population’: Comparison of the study population’s characteristics with local and national data. Click here for file

Additional file 3 Imputation of distances and times, and calculation of transport CO_2_ emissions. Click here for file

Additional file 4 **‘Predictors of CO**_**2**_**emissions for different journey purposes’: Table showing associations between health/activity characteristics and transport CO**_**2**_**emissions for different journey purposes.** Click here for file
