# Supplementary material for: Associations of health, physical activity and weight status with motorised travel and transport carbon dioxide emissions: a cross-sectional, observational study
Source: Environ Health. 2012 Aug 3;11:52. doi: 10.1186/1476-069X-11-52 (PMC3536622; doi:10.1186/1476-069X-11-52)
Supplement: Additional file 1 — ‘Survey Questionnaire’: Blank copy of survey questionnaire. [file 1476-069X-11-52-S1.pdf]

SECTION

A

About your local area

1. Firstly, we'd like to ask you about the **neighbourhood where you live**. By *neighbourhood* we mean the area that you could walk to in **10–15 minutes from your home**. How much do you agree with the following statements about your neighbourhood? **(Tick one box per row.)**

	STRONGLY AGREE	SOMEWHAT AGREE	NEITHER AGREE NOR DISAGREE	SOMEWHAT DISAGREE	STRONGLY DISAGREE
a. Walking is unsafe because of the traffic.	☐	☐	☐	☐	☐
b. Cycling is unsafe because of the traffic.	☐	☐	☐	☐	☐
c. There are no convenient routes for walking and cycling.	☐	☐	☐	☐	☐
d. There are not enough safe places to cross roads.	☐	☐	☐	☐	☐
e. The area is unsafe because of the level of crime or anti-social behaviour.	☐	☐	☐	☐	☐
f. The area is generally free from litter or graffiti.	☐	☐	☐	☐	☐
g. There are places to walk or cycle to (e.g. shops, restaurants, leisure facilities).	☐	☐	☐	☐	☐
h. There are open spaces (e.g. parks, sports fields or beaches).	☐	☐	☐	☐	☐
i. There are pavements suitable for walking.	☐	☐	☐	☐	☐
j. There are special lanes, routes or paths for cycling.	☐	☐	☐	☐	☐
k. There are many road junctions.	☐	☐	☐	☐	☐
l. There are many different routes for walking and cycling so I don't have to go the same way every time.	☐	☐	☐	☐	☐
m. The area is pleasant for walking or cycling.	☐	☐	☐	☐	☐

2. Now we would like to ask you about **travelling between Penarth and Cardiff Bay**. To what extent do you agree with the following statements? **(Tick one box per row.)**

	STRONGLY AGREE	SOMEWHAT AGREE	NEITHER AGREE NOR DISAGREE	SOMEWHAT DISAGREE	STRONGLY DISAGREE
a. Walking is unsafe because of the traffic.	☐	☐	☐	☐	☐
b. Cycling is unsafe because of the traffic.	☐	☐	☐	☐	☐
c. The level of crime or anti-social behaviour means walking or cycling is unsafe.	☐	☐	☐	☐	☐
d. There are pavements suitable for walking.	☐	☐	☐	☐	☐
e. There are special lanes, routes or paths for cycling.	☐	☐	☐	☐	☐
f. The routes for walking and cycling are generally well lit at night.	☐	☐	☐	☐	☐
g. The routes are pleasant for walking or cycling.	☐	☐	☐	☐	☐

About your travel

SECTION B

We are interested in your views about

walking and cycling to travel from place to place.

By *walking and cycling to travel*, we mean any walking and cycling you do to get to places. For example, going to work, going out to get lunch, coming home from work, going shopping, going to the bus or railway station, visiting friends, or escorting someone else (for example, taking a child to school). ⚠ **We do not** mean any walking or cycling you do for recreation, health or fitness—we will ask you about this later.

PLEASE COMPLETE THESE QUESTIONS EVEN IF YOU DON'T DO MUCH WALKING OR CYCLING.

3. Think about **walking to travel** from place to place.
How much do you agree with the following statements? **(Tick one box per row.)**

	STRONGLY AGREE	SOMEWHAT AGREE	NEITHER AGREE NOR DISAGREE	SOMEWHAT DISAGREE	STRONGLY DISAGREE
a. Walking to travel from place to place is something I do automatically without really thinking about it.	☐	☐	☐	☐	☐
b. It is beneficial for me to walk for travel.	☐	☐	☐	☐	☐
c. Walking for travel is enjoyable.	☐	☐	☐	☐	☐
d. The people in my life whose opinions I value most would approve of me walking for travel.	☐	☐	☐	☐	☐
e. Most people who are important to me walk for travel.	☐	☐	☐	☐	☐
f. It is possible for me to walk for travel.	☐	☐	☐	☐	☐
g. It is mostly up to me whether I walk for travel.	☐	☐	☐	☐	☐
h. I intend to do more walking for travel over the coming months.	☐	☐	☐	☐	☐
i. I see people in my neighbourhood walking for travel.	☐	☐	☐	☐	☐
j. Over the last 12 months I have done more walking for travel.	☐	☐	☐	☐	☐

Continued

4. Think about **cycling to travel** from place to place.
How much do you agree with the following statements? **(Tick one box per row.)**

	STRONGLY AGREE	SOMEWHAT AGREE	NEITHER AGREE NOR DISAGREE	SOMEWHAT DISAGREE	STRONGLY DISAGREE
a. Cycling to travel from place to place is something I do automatically without really thinking about it.	☐	☐	☐	☐	☐
b. It is beneficial for me to cycle for travel.	☐	☐	☐	☐	☐
c. Cycling for travel is enjoyable.	☐	☐	☐	☐	☐
d. The people in my life whose opinions I value most would approve of me cycling for travel.	☐	☐	☐	☐	☐
e. Most people who are important to me cycle for travel.	☐	☐	☐	☐	☐
f. It is possible for me to cycle for travel.	☐	☐	☐	☐	☐
g. It is mostly up to me whether I cycle for travel.	☐	☐	☐	☐	☐
h. I intend to do more cycling for travel over the coming months.	☐	☐	☐	☐	☐
i. I see people in my neighbourhood cycling for travel.	☐	☐	☐	☐	☐
j. Over the last 12 months I have done more cycling for travel.	☐	☐	☐	☐	☐

The next set of questions asks about the **vehicles** you have access to and the vehicles you actually use.

5. How many of the following vehicles are kept in your household? (Include all vehicles kept overnight.)

	WRITE IN NUMBER	IF ZERO, TICK HERE		WRITE IN NUMBER	IF ZERO, TICK HERE
Bicycles for adults		☐	Private cars and vans		☐
Bicycles for children		☐	Motorcycles		☐
Company cars and vans		☐			

6. Please tell us about the **cars and vans** you **actually use**. These may be among the cars or vans from the previous question, but they could also include other vehicles owned by friends or family.
If you do not use any cars or vans please tick here ☐ and go to question 8.

VEHICLE NO.	MAKE AND MODEL	FUEL TYPE	ENGINE SIZE	AGE (IN YEARS)	HOW MUCH HAVE YOU SPENT ON FUEL FOR THIS VEHICLE IN THE **LAST seven (7) DAYS**?
1		☐ PETROL ☐ DIESEL ☐ OTHER (PLEASE SPECIFY): __________	☐ LESS THAN 1.4 LITRES ☐ 1.4–2.0 LITRES ☐ MORE THAN 2.0 LITRES		£ TICK HERE IF £0 ☐
2		☐ PETROL ☐ DIESEL ☐ OTHER (PLEASE SPECIFY): __________	☐ LESS THAN 1.4 LITRES ☐ 1.4–2.0 LITRES ☐ MORE THAN 2.0 LITRES		£ TICK HERE IF £0 ☐
3		☐ PETROL ☐ DIESEL ☐ OTHER (PLEASE SPECIFY): __________	☐ LESS THAN 1.4 LITRES ☐ 1.4–2.0 LITRES ☐ MORE THAN 2.0 LITRES		£ TICK HERE IF £0 ☐
4		☐ PETROL ☐ DIESEL ☐ OTHER (PLEASE SPECIFY): __________	☐ LESS THAN 1.4 LITRES ☐ 1.4–2.0 LITRES ☐ MORE THAN 2.0 LITRES		£ TICK HERE IF £0 ☐

7. Which of these **vehicles did you use most** over the **last seven (7) days**?
(Please refer to question 6 for the **vehicle number**.)

We'd now like to ask about

your journeys in the last seven days

Please include all the journeys you made however long or short, using any method of transport, not just walking and cycling. **Four points to note**
① A return journey counts as one journey. For example, if you travelled to work and back five (5) times, this counts as five (5) journeys. ② Where a return journey involves a number of purposes, please give the **main** purpose. ③ Include all methods of travel you used as part of a journey (e.g. walking to a bus stop and then catching the bus). ④ If you spent time waiting for public transport please include this within the public transport journey time. Here is an example:

FIVE (5) RETURN JOURNEYS TO WORK

10 minutes (each way) x 5 (return journeys) = 100 minutes (1 hour 40 minutes)
0.5 miles (each way) x 5 (return journeys) = 5 miles

20 minutes (each way) x 5 (return journeys) = 200 minutes (3 hours 20 minutes)
25 miles (each way) x 5 (return journeys) = 250 miles

PLEASE COMPLETE THESE QUESTIONS EVEN IF YOU DON'T TRAVEL AROUND VERY MUCH IN GENERAL OR YOU DO NOT DO VERY MUCH WALKING OR CYCLING.

8. Think about your **journeys to and from work**.
(e.g. travel to and from your place of work, accompanying your spouse to and from their work).

a. How often did you make such a journey over the **last seven (7) days**? ☐ TIMES ☐ **IF ZERO TIMES, TICK HERE AND GO TO QUESTION 9.**

b. How much time in total over the last seven (7) days did you spend travelling **to and from work** by:

	HOURS	MINUTES
Walking		
Cycle		
Bus		
Train		
Car, as a driver		
Car, as a passenger		
Other (please specify): ___________		

c. How far did you travel in total over the last seven (7) days **to and from work** by:

	MILES
Walking	
Cycle	
Bus	
Train	
Car, as a driver	
Car, as a passenger	
Other (please specify): ___________	

Continued

9. Think about your **business journeys**, by which we mean any journeys in the course of your work or on employer's business (e.g. travel to and from meetings, making deliveries, etc.)

a. How often did you make such a journey over the **last seven (7) days**? ☐ TIMES ☐ IF ZERO TIMES, TICK HERE AND GO TO QUESTION 10.

b. How much time in total over the last seven (7) days did you spend travelling **on business journeys** by:

	HOURS	MINUTES
Walking		
Cycle		
Bus		
Train		
Car, as a driver		
Car, as a passenger		
Other (please specify): ____________		

c. How far did you travel in total over the last seven (7) days **on business journeys** by:

	MILES
Walking	
Cycle	
Bus	
Train	
Car, as a driver	
Car, as a passenger	
Other (please specify): ____________	

10. Think about your **journeys to and from a place of study** (e.g. travel to and from your university or college) or **to and from school** (e.g. if you accompany a child to and from school).

a. How often did you make such a journey over the **last seven (7) days**? ☐ TIMES ☐ IF ZERO TIMES, TICK HERE AND GO TO QUESTION 11.

b. How much time in total over the last seven (7) days did you spend travelling **to and from a place of study** or **school** by:

	HOURS	MINUTES
Walking		
Cycle		
Bus		
Train		
Car, as a driver		
Car, as a passenger		
Other (please specify): ____________		

c. How far did you travel in total over the last seven (7) days **to and from a place of study** or **school** by:

	MILES
Walking	
Cycle	
Bus	
Train	
Car, as a driver	
Car, as a passenger	
Other (please specify): ____________	

11. Think about your **journeys for shopping and personal business**
(e.g. food shopping, non-food shopping, window-shopping, visiting a doctor, bank, solicitor or estate agents,visiting a relative in hospital, or accompanying someone else to a doctor, hospital etc.).

a. How often did you make such a journey over the **last seven (7) days**? [ ] TIMES ☐ IF ZERO TIMES, TICK HERE AND GO TO QUESTION 12.

b. How much time in total over the last seven (7) days did you spend travelling for **shopping and personal business** by:

	HOURS	MINUTES
Walking		
Cycle		
Bus		
Train		
Car, as a driver		
Car, as a passenger		
Other (please specify): ___________		

c. How far did yo travel in total over the last seven (7) days for **shopping and personal business** by:

	MILES
Walking	
Cycle	
Bus	
Train	
Car, as a driver	
Car, as a passenger	
Other (please specify): ____________	

12. Think about your **journeys to visit friends and relatives** and for **other social activities**.
(e.g. a journey to and from the cinema or other entertainment facilities).

a. How often did you make such a journey over the **last seven (7) days**? [ ] TIMES ☐ IF ZERO TIMES, TICK HERE AND GO TO QUESTION 13.

b. How much time in total over the last seven (7) days did you spend travelling to **visit friends or relatives** or for **other social activities** by:

	HOURS	MINUTES
Walking		
Cycle		
Bus		
Train		
Car, as a driver		
Car, as a passenger		
Other (please specify): ___________		

c. How far did you travel in total over the last seven (7) days to **visit friends or relatives** or for **other social activities** by:

	MILES
Walking	
Cycle	
Bus	
Train	
Car, as a driver	
Car, as a passenger	
Other (please specify): ____________	

Continued

SECTION

C

About your recreation and leisure-time activities

We are interested in your views about

walking and cycling for recreation.

By *walking and cycling for recreation*, we mean any walking and cycling you have done for leisure, health or fitness including, for example, walking or cycling in parks or along trails or walking the dog. ⚠ **We do not** mean walking or cycling you may do for the primary purpose of travel to get from place to place.

PLEASE COMPLETE THESE QUESTIONS EVEN IF YOU DON'T DO MUCH WALKING OR CYCLING.

13. Think about **walking for recreation**. How much do you agree with the following statements? **(Tick one box per row.)**

	STRONGLY AGREE	SOMEWHAT AGREE	NEITHER AGREE NOR DISAGREE	SOMEWHAT DISAGREE	STRONGLY DISAGREE
a. Walking for recreation is something I do automatically without really thinking about it.	☐	☐	☐	☐	☐
b. It is beneficial for me to walk for recreation.	☐	☐	☐	☐	☐
c. Walking for recreation is enjoyable.	☐	☐	☐	☐	☐
d. The people in my life whose opinions I value most would approve of me walking for recreation.	☐	☐	☐	☐	☐
e. Most people who are important to me walk for recreation.	☐	☐	☐	☐	☐
f. It is possible for me to walk for recreation.	☐	☐	☐	☐	☐
g. It is mostly up to me whether I walk for recreation.	☐	☐	☐	☐	☐
h. I intend to do more recreational walking over the coming months.	☐	☐	☐	☐	☐
i. I see people in my neighbourhood walking for recreation.	☐	☐	☐	☐	☐
j. Over the last 12 months I have done more walking for recreation.	☐	☐	☐	☐	☐

14. In the **last seven (7) days**, did you do any **walking** for recreation, health or fitness?

☐ YES
☐ NO (IF NO GO TO Q15.)

a. In the **last seven (7) days**, how many times did you **walk** for recreation, health or fitness (including walking your dog)? ☐ TIMES

b. Please estimate the total time you spent **walking** for recreation, health or fitness in the **last seven (7) days** (e.g. 2 times x 20 minutes = 40 minutes).

HOURS ☐ MINUTES ☐

15. Think about **cycling for recreation**. How much do you agree with the following statements? **(Tick one box per row.)**

	STRONGLY AGREE	SOMEWHAT AGREE	NEITHER AGREE NOR DISAGREE	SOMEWHAT DISAGREE	STRONGLY DISAGREE
a. Cycling for recreation is something I do automatically without really thinking about it.	☐	☐	☐	☐	☐
b. It is beneficial for me to cycle for recreation.	☐	☐	☐	☐	☐
c. Cycling for recreation is enjoyable.	☐	☐	☐	☐	☐
d. The people in my life whose opinions I value most would approve of me cycling for recreation.	☐	☐	☐	☐	☐
e. Most people who are important to me cycle for recreation.	☐	☐	☐	☐	☐
f. It is possible for me to cycle for recreation.	☐	☐	☐	☐	☐
g. It is mostly up to me whether I cycle for recreation.	☐	☐	☐	☐	☐
h. I intend to do more recreational cycling over the coming months.	☐	☐	☐	☐	☐
i. I see people in my neighbourhood cycling for recreation.	☐	☐	☐	☐	☐
j. Over the last 12 months I have done more cycling for recreation.	☐	☐	☐	☐	☐

16. In the **last seven (7) days**, did you do any **cycling** for recreation, health or fitness?

☐ YES
☐ NO (IF NO GO TO Q17.)

a. In the **last seven (7) days**, how many times did you **cycle** for recreation, health or fitness? ☐ TIMES

b. Please estimate the total time you spent **cycling** for recreation, health or fitness in the **last seven (7) days**. (e.g. 2 times x 20 minutes = 40 minutes). HOURS ☐ MINUTES ☐

The next set of questions is about **other leisure-time physical activities** that you have done in the **last seven (7) days**, besides what you have already mentioned. ⚠ Please do not include any walking or cycling in answering the questions below.

17. In the **last seven (7) days**, did you do any vigorous-intensity, leisure-time physical activities like jogging, aerobics or competitive tennis? **Do not** include walking or cycling or moderate-intensity physical activities. Vigorous-intensity physical activities make you breathe harder or puff and pant.

☐ YES
☐ NO (IF NO GO TO Q18.)

a. In the **last seven (7) days**, how many times did you do vigorous-intensity, leisure-time physical activities which made you breathe harder or puff and pant? ☐ TIMES

b. Please estimate the total time you spent doing vigorous-intensity, leisure-time physical activities in the **last seven (7) days**. HOURS ☐ MINUTES ☐

18. Apart from what you have already mentioned, in the **last seven (7) days**, have you done any other moderate-intensity, leisure-time physical activities like gentle swimming, social tennis, golf or heavy gardening? Moderate intensity physical activities make you breathe somewhat harder than normal.

☐ YES
☐ NO (IF NO GO TO Q19.)

a. In the **last seven (7) days**, how many times did you do moderate-intensity, leisure-time physical activities which made you breathe somewhat harder than normal? ☐ TIMES

b. Please estimate the total time you spent doing moderate-intensity, leisure-time physical activities in the **last seven (7) days**. HOURS ☐ MINUTES ☐

Continued

SECTION

D

About your local pedestrian and cycling routes

You may be aware that in the past year a new bridge for pedestrians and cyclists has been opened over the River Ely in Cardiff Bay. This is known locally as the Pont-y-Werin Bridge "The People's Bridge".

19. Had you heard of the People's Bridge before completing this survey?

☐ YES
☐ NO

20. Do you use the People's Bridge?

☐ YES
☐ NO (IF NO GO TO Q23.)

21. Think about the People's Bridge ...and **walking**. Do you **walk** across the People's Bridge ...?

	YES	NO
a. On your way to or from work.	☐	☐
b. For business-related journeys.	☐	☐
c. On your way to or from a place of study (e.g. college/university).	☐	☐
d. To get to the shops or for personal business (e.g. visiting a doctor, bank, solicitor or estate agents).	☐	☐
e. On your way to visit friends and relatives or to do other social activities.	☐	☐
f. For recreation, health or fitness.	☐	☐

22. Think about the People's Bridge ...and **cycling**. Do you **cycle** across the People's Bridge ...?

	YES	NO
a. On your way to or from work.	☐	☐
b. For business-related journeys.	☐	☐
c. On your way to or from a place of study (e.g. college/university).	☐	☐
d. To get to the shops or for personal business (e.g. visiting a doctor, bank, solicitor or estate agents).	☐	☐
e. On your way to visit friends and relatives or to do other social activities.	☐	☐
f. For recreation, health or fitness.	☐	☐

SECTION **E**

About your work or place of study

23. Think about the work you do. Which of these best describes your situation at present? **(Tick one only.)**

- Doing paid work full-time ☐
- Doing paid work part-time ☐
- Full-time student ☐

GO TO QUESTION 24A

- Unemployed ☐
- Retired ☐
- Looking after home or family ☐
- Permanently sick or disabled ☐
- Other (please specify) ______________________ ☐

GO TO SECTION F

24a. What is the postcode of your **main** place of work or study? ☐☐☐☐ ☐☐☐

24b. If you do not know the postcode, please give the address of your place of work or study

25. Please **tick** the option that **best** corresponds with your work or study. **(Tick one only.)**

Sedentary occupation
You spend most of your time sitting
(e.g. in an office, driving a vehicle). ☐

Standing occupation
You spend most of your time standing or walking.
However, your work does not require intense physical effort
(e.g. shop assistant, hairdresser, guard). ☐

Manual work
This involves some physical effort including
handling of heavy objects and use of tools
(e.g. plumber, electrician, carpenter). ☐

Heavy manual work
This implies very vigorous physical activity
including handling of very heavy objects
(e.g. dock worker, miner, bricklayer, construction worker). ☐

Continued

SECTION

F

About you and your household

26. Are you male or female? **(Tick one only.)** ☐ MALE ☐ FEMALE

27. How old are you? ☐ YEARS

28. How much do you weigh in light indoor clothes? ☐ STONES ☐ LBS or ☐ KG

29. How tall are you without shoes on? ☐ FEET ☐ INCHES or ☐ CM

30. Do you have any long-term illness, health problem or disability which limits your daily activities or the work you can do? (Include problems which are due to old age.) ☐ YES ☐ NO

31. Would you say that for someone of your age your own health in general is… **(Tick one only.)**

- Excellent ☐
- Good ☐
- Fair ☐
- Poor ☐

32. Which of the following groups do you consider you belong to? **(Tick one only.)**

- White ☐
- Mixed ethnic group ☐
- Asian or Asian British ☐
- Black or Black British ☐
- Other (Please specify): _________________ ☐

33. What is your highest educational qualification? **(Tick one only.)**

- Degree, NVQ4, NVQ5 (or equivalent) ☐
- BTEC (Higher), BEC (Higher), TEC (Higher), HNC, HND (or equivalent) ☐
- GCE ‘A’ Level, NVQ3, Scottish Higher (or equivalent) ☐
- BTEC (National), BEC (National), TEC (National), ONC, OND (or equivalent) ☐
- GCSE Grades A to C, GCE 'O' Level, CSE Grade 1, NVQ2 (or equivalent) ☐
- Other qualifications ☐
- No formal qualifications ☐

34. What is the postcode of your home? ☐☐☐☐ ☐☐☐

35. How long have you lived in your current home? ☐ YEARS ☐ MONTHS

36. How many people, other than you, live in your household?
We mean people who have your accommodation as their only or main residence, and who either share at least one meal a day with you or share the living accommodation (living room or sitting room) with you. **(Write in number.)**

Children aged under 5	☐	IF NONE, TICK HERE. ☐
Children aged between 5 and 15	☐	IF NONE, TICK HERE. ☐
Adults aged 16 and over (do not include yourself)	☐	IF NONE, TICK HERE. ☐

37. Does your household own or rent its accommodation? **(Tick one only.)**

- Rents it from the council, a housing association or a charity ☐
- Rents it from a private landlord or letting agency ☐
- Partly owns it and partly rents it (shared ownership) ☐
- Owns it (including buying with a mortgage) ☐
- Other ☐

38. What is your total household income from all sources before tax? **(Tick one only.)**

- Up to £10,000 ☐
- £10,001–£20,000 ☐
- £20,001–£30,000 ☐
- £30,001–£40,000 ☐
- £40,001–£50,000 ☐
- More than £50,000 ☐
- Don’t know ☐

39. Are you aware of, or taking part in, any projects in your area relating to walking and cycling? ☐ YES ☐ NO

If yes, please specify: ______________________________

____

____

____

40. Please enter the date on which you are completing this survey.

DAY ☐☐ MONTH ☐☐ YEAR ☐☐

41. Are there any other comments you would like to add?

____

____

____
