# Supplementary material for: Associations of health, physical activity and weight status with motorised travel and transport carbon dioxide emissions: a cross-sectional, observational study
Source: Environ Health. 2012 Aug 3;11:52. doi: 10.1186/1476-069X-11-52 (PMC3536622; doi:10.1186/1476-069X-11-52)
Supplement: Additional file 2 — ‘Representativeness of study population’: Comparison of the study population’s characteristics with local and national data. [file 1476-069X-11-52-S2.doc]

Table 1: Comparison of the characteristics of the study population (N= 3,463) with those of the general population

| **Domain** | **Variable** | **Level** | **Study sample, unweighted (%)** | **Study sample, weighted† (%)** | **General population (%)** | **Comparison population** |
| --- | --- | --- | --- | --- | --- | --- |
| Demo- | Sex | Female | 55 | 51 | 51 | Local: Office |
| graphic |  | Male | 45 | 49 | 49 | for National |
|  | Age (years) | 18-29 | 15 | 26 | 26 | Statistics 2010 ^a^ |
|  |  | 30-49 | 31 | 35 | 35 |  |
|  |  | 50-64 | 32 | 22 | 22 |  |
|  |  | 65+ | 22 | 17 | 17 |  |
|  | Ethnicity | White | 95 | 94 | 94 | Local: Census |
|  |  | Non-White | 5 | 6 | 6 | 2001 ^b^ |
|  | Any child | No | 80 | 76 | 60 |  |
|  | under 16 | Yes | 20 | 24 | 40 |  |
|  | Urban/rural | Urban | 95 | 96 | 94 |  |
|  | status | Rural | 5 | 4 | 6 |  |
| Socio- | Highest | Degree | 41 | 44 | 26 |  |
| economic | educational | A-level | 18 | 19 | 11 |  |
|  | qualification | GCSE | 19 | 17 | 16 |  |
|  |  | None or other | 23 | 19 | 46 |  |
|  | Tenure | Owns | 76 | 71 | 70 |  |
|  |  | Rents | 24 | 29 | 31 |  |
|  | Employment | Employed | 56 | 60 | 64 |  |
|  | status | Unemployed | 2 | 3 | 3 |  |
|  |  | Student | 6 | 9 | 6 |  |
|  |  | Other economically inactive | 36 | 28 | 27 |  |
| Health | Weight status | Normal/underweight | 51 | 54 | 39 | National: Health |
|  |  | Overweight | 35 | 32 | 38 | survey for |
|  |  | Obese | 14 | 13 | 23 | England 2009 ^c^ |
|  | General | Excellent/good | 78 | 77 | 63 | Local: Census |
|  | health | Fair/poor | 22 | 23 | 37 | 2001 ^b^ |
|  | Long-term | No | 78 | 81 | 79 |  |
|  | limiting illness | Yes | 22 | 19 | 21 |  |
| Travel | Cars per adult | No cars | 15 | 16 | 20 |  |
|  | in household | <1 car per adult | 37 | 37 | 35 |  |
|  |  | ≥1 cars per adult | 48 | 47 | 44 |  |
|  | Main mode to | Car | 70 | 69 | 73 |  |
|  | work (mode | Public transport | 12 | 12 | 10 |  |
|  | involving the | Walk | 11 | 11 | 13 |  |
|  | greatest distance) | Cycle | 7 | 8 | 4 |  |
|  | Percentage travel | Car | 79 | 78 | 78 | National: |
|  | distance covered | Bus or train | 14 | 15 | 14 | National Travel |
|  | by different modes | Walk | 3 | 3 | 3 | Survey, 2010 ^d^ |
|  |  | Cycle | 2 | 2 | 1 |  |
|  |  | Other modes | 2 | 2 | 4 |  |

† Study sample weighted by age and sex, as in our main paper.

^a^ Office for National Statistics mid 2010 population estimates [1], percentages calculated by authors. We included all adult residents (aged ≥16years) living in the three local authorities from which we drew our study samples, giving equal weighting to each local authority.

^b^ Census 2001 5% sample in Small Area Microdata [2], percentages calculated by authors. We included all adult residents (aged >20years) living in private households in the three local authorities from which we drew our study samples, giving equal weighting to each local authority. To ensure comparability, we also restricted our study sample to those ages 20 or more (97% of sample) when making comparisons with the census data.

^c^ Health Survey for England 2009, adult sample [3]

^d^ National Travel Survey 2010 [4].

**References**
