# Supplementary material for: Associations of health, physical activity and weight status with motorised travel and transport carbon dioxide emissions: a cross-sectional, observational study
Source: Environ Health. 2012 Aug 3;11:52. doi: 10.1186/1476-069X-11-52 (PMC3536622; doi:10.1186/1476-069X-11-52)
Supplement: Additional file 3 — Imputation of distances and times, and calculation of transport CO2 emissions. [file 1476-069X-11-52-S3.doc]

**Imputation of missing distance or time data**

Table 1: Mean speed (miles/hour) by mode, purpose and Site, as used for imputation of missing time or distance values

| **Purpose** | **Mode** | **Southampton** | **Cardiff** | **Kenilworth** | **Average across all sites** |
| --- | --- | --- | --- | --- | --- |
| **Commute** | **Walk** | 3.0 | 3.2 | 3.3 | 3.2 |
|  | **Cycle** | 8.8 | 8.7 | 10.8 | 9.2 |
|  | **Bus** | 10.4 | 9.7 | 10.2 | 10.2 |
|  | **Train** | 38.7 | 26.3 | 50.2 | 35.2 |
|  | **Car driver** | 23.1 | 23.2 | 27.1 | 24.7 |
|  | **Car passenger** | 19.1 | 19.2 | 21.2 | 19.8 |
| **Education** | **Walk** | 2.9 | 2.8 | 3.4 | 3.0 |
|  | **Cycle** | 8.0 | 6.7 | 8.4 | 7.9 |
|  | **Bus** | 14.7 | 14.1 | 9.2 | 13.4 |
|  | **Train** | 24.0 | 27.0 | 43.9 | 30.5 |
|  | **Car driver** | 14.3 | 16.3 | 19.7 | 17.3 |
|  | **Car passenger** | 17.9 | 19.7 | 23.9 | 20.8 |
| **Business** | **Walk** | 2.9 | 3.4 | 3.2 | 3.1 |
|  | **Cycle** | 8.3 | 7.2 | 13.3 | 9.4 |
|  | **Bus** | 8.9 | 9.2 | 11.1 | 9.6 |
|  | **Train** | 43.7 | 50.6 | 50.9 | 48.8 |
|  | **Car driver** | 29.2 | 29.0 | 30.8 | 29.8 |
|  | **Car passenger** | 23.8 | 26.4 | 25.7 | 25.5 |
| **Shopping** | **Walk** | 2.8 | 2.9 | 3.0 | 2.9 |
|  | **Cycle** | 7.6 | 7.2 | 8.0 | 7.6 |
|  | **Bus** | 8.3 | 11.2 | 11.0 | 10.2 |
|  | **Train** | 26.8 | 20.4 | 35.7 | 26.2 |
|  | **Car driver** | 17.3 | 17.5 | 20.4 | 18.7 |
|  | **Car passenger** | 15.8 | 17.3 | 20.7 | 18.2 |
| **Social** | **Walk** | 3.2 | 3.0 | 3.2 | 3.1 |
|  | **Cycle** | 7.8 | 6.7 | 7.5 | 7.4 |
|  | **Bus** | 10.4 | 13.7 | 13.7 | 12.3 |
|  | **Train** | 35.8 | 28.0 | 46.6 | 35.9 |
|  | **Car driver** | 24.6 | 23.0 | 27.3 | 25.1 |
|  | **Car passenger** | 20.3 | 22.2 | 25.5 | 22.9 |

**Calculation of transport CO_2_ emissions**

As described in the main text we used a detailed seven-day recall instrument to measure past-week travel behaviour across a range of modes. For travel by bus, train, and other non-car modes (taxi, ferry, underground, motorcycle and mobility scooter), we calculated the total distance travelled in the past week and multiplied this by mode-specific, average emissions factors included in UK Government 2010 guidelines for greenhouse gas reporting . We excluded the flights reported by 18 participants (0.5%) because our survey instrument was not designed to capture CO_2_ emissions from air travel and because, unlike for land travel, past-week air travel is unlikely to be a good proxy for average air travel.

For cars and vans we further calibrated the process of estimating CO_2_ emissions by using participants’ reports of their time spent travelling to calculate average travel speed as a proxy for road types (e.g. urban, rural, motorway). This allowed us to apply the speed-emissions factors underlying the National Atmospheric Emissions Inventory . These speed-emissions curves are polynomial functions of emissions as a function of average speed, vehicle type (car or van), fuel type and propulsion technology (petrol ICE, diesel ICE, LPG ICE, petrol HEV), engine size (<1.4 litres, 1.4-2 litres, >2 litres) and vehicle age. The ‘most used vehicle’ reported by the participants was taken as the reference vehicle for the emissions analysis. Where one or more vehicle details were omitted, the average of the unreported variable was taken as the basis for emissions factors. For example, some respondents did not report engine size while providing details on fuel type and age. In these (few) cases we used the 2010 fleet average of petrol and diesel car emissions factors.

Multiplying total distance travelled by these speed-emissions factors gave us an estimate of the total ‘hot’ emissions for each vehicle when the engine was warmed up. As a final adjustment to this figure, we used the total number of reported trips by each participant to estimate the number of ‘cold’ starts, as cold starts generate excess emissions (i.e. over and above the ‘hot’ emissions) due to suboptimal fuel combustion. Excess cold start emissions were calculated as a function of the ambient air temperature, average trip length and the share of the trip length running ‘cold’.

In the absence of detailed data on the shared use of cars, previous studies have allocated all CO_2_ emissions to the driver . The advantage of this method is that by not allocating full emissions to both drivers and passengers, it avoids overestimating the total CO_2_ contribution from car travel. One disadvantage of this approach, however, would be to render our path analysis (Figure 1 of main text) inconsistent: because we wanted to include passenger car travel within our measure of motorised travel distance we felt that this motorised travel should also contribute to CO_2_ emissions. Another disadvantage is that assigning zero emissions to passenger travel by car is arguably inconsistent with the fact that we did assign CO_2_ emissions for passenger travel by public transport. Finally we were concerned that the driver-only approach might introduce a spurious association with CO_2_ emissions if a particular health characteristic was not associated with levels of car use but was associated with whether an individual chose to do the driving themselves or asked their partner to do it.

We therefore explored using an alternative approach which aimed to divide emissions between car drivers and car passengers as follows. First we calculated emissions under a ‘worst case’ scenario, assuming car drivers were alone in their vehicle (i.e. receiving full emissions) and that car passengers were in a car with only them and the driver (i.e. receiving half-emissions). We then scaled both sets of emissions downwards by the ratio of [driver CO_2_ emissions/(driver + passenger CO_2_ emissions)], calculating this ratio separately for each mode (ratios 0.94 for commuting to work, 0.89 for commuting to education, 0.96 for business travel, 0.86 for shopping/personal business, and 0.85 for social/leisure journeys). We did this in order to avoid overestimating the total CO_2_ contribution from car travel, in recognition of the fact that some fraction of drivers will have had passengers in the car (fraction estimated from the group level data) and some fraction of passengers will have been in cars with other passengers as well (fraction unknown but assumed to be the same).

This driver/passenger approach yielded very similar estimates of total CO_2_ emissions to the ‘driver-only’ approach (Pearson’s correlation 0.91) and very similar substantive findings. The only minor exception was that while the association between poor general health and CO_2_ was attenuated to the null using the driver/passenger approach (regression coefficient -0.16, 95%CI -0.35, 0.04: see multivariable model 2 in Table 2 of main text) it remained weakly significant using the driver-only approach (-0.28, 95%CI -0.53, -0.03). This small difference seemed to reflect the fact that poor general health was associated with spending a higher percentage of car travel time as a passenger (45% time as a passenger for those with poor general health vs. 25% for those with good/excellent health; p=0.003 for difference in multivariable analyses). We interpreted this as reflecting individuals with poor health asking other individuals (e.g. their partner) to drive when going out on joint trips, and this seemed to us the sort of spurious association with CO_2_ emissions that we had sought to avoid. We therefore use the driver/passenger approach throughout the main text.

**References**
