# Supplementary material for: Associations of health, physical activity and weight status with motorised travel and transport carbon dioxide emissions: a cross-sectional, observational study
Source: Environ Health. 2012 Aug 3;11:52. doi: 10.1186/1476-069X-11-52 (PMC3536622; doi:10.1186/1476-069X-11-52)
Supplement: Additional file 4 — ‘Predictors of CO2emissions for different journey purposes’: Table showing associations between health/activity characteristics and transport CO2emissions for different journey purposes. [file 1476-069X-11-52-S4.doc]

**Additional file 4, Table 1: Health and physical activity predictors of transport CO_2_ emissions from different types of journey (n=3463)**

|  |  | **Commuting or in the course of business**  **(57% of all CO_2_ emissions)** | | **Shopping or personal business**  **(19% of all CO_2_ emissions)** | | **Social visits or leisure activities**  **(24% of all CO_2_ emissions)** | |
| --- | --- | --- | --- | --- | --- | --- | --- |
|  |  | **Median kgCO_2_/ week** | **Multivariable regression coefficient (95%CI)** | **Median kgCO_2_/ week** | **Multivariable regression coefficient (95%CI)** | **Median kgCO_2_/ week** | **Multivariable regression coefficient (95%CI)** |
| Body mass | Normal | 3.2 | 0* | 2.2 | 0*** | 2.0 | 0 |
| Index | Overweight | 5.2 | .09 (.02, .15) | 3.6 | .17 (.08, .25) | 2.7 | .07 (-.01, .16) |
|  | Obese | 3.7 | .09 (-.01, .18) | 3.6 | .21 (.09, .32) | 2.0 | -.01 (-.14, .11) |
| General | Excellent/good | 5.2 | 0 | 3.0 | 0 | 2.5 | 0 |
| Health | Fair | 0.3 | -.03 (-.12, .05) | 2.6 | -.01 (-.12, .09) | 1.4 | -.02 (-.12, .09) |
|  | Poor | 0.0 | -.06 (-.20, .08) | 1.9 | .01 (-.20, .22) | 0.5 | -.12 (-.29, .06) |
| Long-term | No | 6.6 | 0 | 2.8 | 0 | 2.3 | 0 |
| illness | Yes | 0.0 | .01 (-.07, .09) | 3.7 | .07 (-.04, .18) | 1.7 | .00 (-.10, .10) |
| Walking | None | 2.8 | 0* | 2.3 | 0** | 1.1 | 0** |
| for recreation | 1-149min | 5.8 | .07 (.00, .14) | 3.1 | .15 (.07, .24) | 2.6 | .12 (.04, .21) |
| in past week | 150-419min | 4.0 | .13 (.05, .21) | 3.3 | .06 (-.05, .17) | 3.1 | .15 (.04, .25) |
|  | ≥420min | 0.0 | .01 (-.13, .14) | 3.3 | .03 (-.12, .18) | 3.7 | .17 (.02, .31) |
| Cycling | None | 3.2 | 0 | 3.0 | 0 | 2.0 | 0 |
| for recreation | 1-149min | 7.2 | .05 (-.08, .18) | 2.3 | -.12 (-.27, .03) | 2.5 | .10 (-.06, .25) |
| in past week | ≥150min | 5.0 | .18 (-.01, .37) | 2.2 | .01 (-.18, .20) | 2.1 | .01 (-.17, .19) |
| Other | None | 1.2 | 0 | 2.4 | 0** | 1.0 | 0*** |
| leisure-time | 1-149min | 6.7 | .07 (.00, .15) | 2.7 | .01 (-.09, .11) | 2.7 | .20 (.10, .30) |
| MVPA | 150-419min | 7.3 | .10 (.02, .18) | 3.3 | .18 (.08, .28) | 3.2 | .25 (.15, .34) |
| in past week | ≥420min | 3.2 | .06 (-.05, .18) | 4.7 | .10 (-.05, .25) | 4.5 | .31 (.17, .46) |
| Walking | None | 7.8 | 0*** | 3.8 | 0 | 2.9 | 0 |
| for transport | 1-149min | 4.8 | -.11 (-.18, -.04) | 2.8 | -.07 (-.16, .02) | 2.2 | -.02 (-.11, .07) |
| in past week | 150-419min | 0.4 | -.27 (-.36, -.19) | 2.3 | -.10 (-.21, .01) | 1.6 | -.10 (-.21, .01) |
|  | ≥420min | 0.0 | -.22 (-.38, -.07) | 1.3 | -.11 (-.27, .06) | 0.4 | -.18 (-.34, -.02) |
| Cycling | None | 4.2 | 0*** | 3.1 | 0* | 2.3 | 0* |
| for transport | 1-149min | 3.0 | -.35 (-.49, -.20) | 1.5 | -.11 (-.26, .03) | 1.2 | -.17 (-.33, .00) |
| in past week | ≥150min | 0.6 | -.60 (-.80, -.41) | 1.0 | -.24 (-.42, -.06) | 1.1 | -.21 (-.38, -.03) |

*p<0.05,**p<0.01,***p<0.001 for heterogeneity. kgCO_2_ = kilograms carbon dioxide, MVPA=moderate-to-vigorous physical activity. Analyses adjust for all variables in columns and in Table 1 of the main text (equivalent to multivariable model 3). The outcomes are standardised log-transformed CO_2_ emissions by journey type
